# 肺癌靶向治疗和免疫治疗药物临床试验现场核查关注点探讨

**DOI:** 10.3779/j.issn.1009-3419.2022.101.32

**Published:** 2022-07-20

**Authors:** 萌 李, 立靖 徐, 秀丽 李, 荣 高

**Affiliations:** 100044 北京，国家药品监督管理局食品药品审核查验中心 Center for Food and Drug Inspection of National Medical Products Administration, Beijing 100044, China

**Keywords:** 肺肿瘤, 靶向治疗, 免疫治疗, 临床试验, 现场核查, 新药研发, Lung neoplasms, Targeted therapy, Immunotherapy, Clinical trials, On-site inspection, New drug research and development

## Abstract

肺癌靶向治疗和免疫治疗药物是当前抗肿瘤新药研发的热点，临床试验逐年增加。本文在梳理2019年-2021年药品注册临床试验现场核查情况的基础上，结合肺癌靶向治疗和免疫治疗药物特点，探讨此类药物的临床试验现场核查关注点，对肺癌临床试验的规范实施提出建议。

## Abstract (en)

As lung cancer targeted therapy and immunotherapy drugs are the current hot spot in the research and development area of new anti-tumor drugs, the amount of clinical trial in this area is increasing year by year. On the basis of combing the on-site inspections of drug registration clinical trials from 2019 to 2021, combined with the characteristics of lung cancer targeted therapy and immunotherapy drugs, this paper discusses the focus of on-site inspection of clinical trials of such drugs, and puts forward suggestions for the compliant implementation of lung cancer clinical trials.

国家癌症中心发布的2022年癌症统计数据显示，我国平均每天有超过1.11万人诊断为新发癌症，有近6, 600人因癌症死亡。其中不论是发病率还是死亡率，肺癌均占首位。自2006年以来，靶向药物研发飞速发展，使肺癌治疗进入精准医疗和个性化治疗阶段，免疫治疗药物为肺癌患者带来了更多的希望。临床试验是探索和确证药物疗效及安全性的关键手段，是药物研发的核心环节。随着药物研发领域新技术的发展，肺癌治疗药物临床试验逐年增加。国家药品监督管理局药品审评中心（以下简称药审中心）临床试验登记平台2016年-2021年登记的以肺癌为适应证的临床试验数量年均增长48.2%。本文梳理了2019年-2021年肺癌靶向药物和免疫治疗药物注册临床试验现场核查情况，结合药物特点，探讨此类药物临床试验现场核查关注点，并对肺癌临床试验的规范实施提出建议。

## 1 肺癌靶向治疗和免疫治疗药物的批准和临床核查情况

自1933年世界第一例肺癌切除术获得成功至今，人类与肺癌的斗争已有近百年的历史。进入21世纪，在肺癌治疗领域突破性的出现了单克隆抗体药物和酪氨酸激酶抑制剂（tyrosine kinase inhibitor, TKI）靶向治疗药物，以及程序性死亡分子-1（programmed cell death 1, PD-1）及其主要配体（programmed cell death ligand 1, PD-L1）单抗等免疫治疗药物。2015年我国批准了贝伐珠单抗注射液用于非小细胞肺癌（non-small cell lung cancer, NSCLC）一线治疗，为肺癌患者带来了新的治疗手段。在表皮生长因子受体（epidermal growth factor receptor, EGFR）-TKI靶向药物领域，我国先后批准了吉非替尼、厄洛替尼、埃克替尼等一代TKI，阿法替尼、达可替尼等二代TKI，甲磺酸奥希替尼、甲磺酸阿美替尼、甲磺酸伏美替尼等三代TKI；针对NSCLC罕见突变，我国先后批准针对间变性淋巴瘤激酶（anaplastic lymphoma kinase, *ALK*）融合基因的塞瑞替尼、阿来替尼、恩沙替尼等，针对间质上皮细胞转化因子（mesenchymal to epithelial transition factor, *MET*）外显子14跳跃突变的赛沃替尼，以及2021年我国首个附条件获批上市的转染重排（rearranged during transfection, RET）激酶抑制剂普拉替尼等靶向治疗药物；另外具有自主知识产权的国产小分子多靶点抗血管生成药物盐酸安罗替尼也获批上市。在免疫检查点抑制剂方面，纳武利尤单抗、帕博利珠单抗、度伐利尤单抗、阿替利珠单抗、信迪利单抗、替雷利珠单抗、舒格利单抗等PD-1/PD-L1单抗品种也不断获批肺癌适应证。

在国家药品监督管理局食品药品审核查验中心（以下简称核查中心）2019年-2021年开展的新药注册临床核查中，按适应证分类，抗肿瘤药物注册临床核查数量占比分别为23.8%、27.3%和28.4%，均居于年度核查任务量榜首且逐年递增。抗肿瘤药物中适应证为肺癌的占比最大，分别为23.3%、25.7%和23.3%，且全部均为肺癌靶向治疗或免疫治疗药物，其中治疗NSCLC的靶向药或免疫治疗药物占93.3%。从试验设计角度统计，76.7%为Ⅲ期临床试验，56.7%为一线治疗，53.3%为联合用药设计，23.3%为单臂试验，30.0%为开放标签试验，23.3%为安慰剂对照试验；以试验主要终点统计，以无进展生存期（progression-free survival, PFS）或客观缓解率（objective response rate, ORR）为主要终点的占86.7%，以总生存期（overall survival, OS）为主要终点的占16.7%；采用中心阅片的占63.3%。

## 2 临床试验现场核查关注点探讨

肺癌靶向治疗和免疫治疗药物临床试验具有抗肿瘤药物临床试验的一般特点，如受试者预后差、耐受性差、试验设计和风险控制参差不齐^[1]^等。同时也具有自身特点，如不同病理和基因分型直接影响药物应答导致入排标准更为严格，不同类型疗效终点数据的获取方式不同，不良反应较多且存在免疫相关不良事件等。现场核查时在执行统一核查标准的前提下，如何结合以上特点开展更具有针对性的核查工作，是本文探讨的重点。

### 2.1 受试者筛选入组方面

受试者筛选入组是开展临床试验的首要环节。现场核查时需逐一核对每一条入选排除标准的符合性证据，查看入组试验的受试者是否均符合试验方案规定的入选与排除标准。

#### 2.1.1 病理和基因检测

病理检测和基因检测是肺癌靶向治疗药物临床试验的关键入选标准。试验设计通常只针对腺癌、鳞癌或非鳞癌中的一种，病理组织类型为混合型的患者一般不能入组试验。NSCLC发病机制与多种基因突变密切相关，存在EGFR、ALK、MET、RET等特定基因改变通常是肺癌靶向药物临床试验的重要入选标准。另外，受试者在入选临床试验时通常需要排除其他已知激活突变。现场核查时需重点关注病理报告、基因检测结果与试验入选标准的符合性，同时关注其他已知突变的检测结果。在基因检测结果的确认方面，需关注检测机构是否与试验方案中的规定一致，如由各研究单位检测或中心实验室检测。

#### 2.1.2 肺癌伴脑转移

肺癌有多发脑转移的特点，24%-40%的晚期NSCLC患者会出现脑转移^[2]^。出于对受试者安全的考虑，肺癌晚期患者较易因发生脑转移而被临床试验排除或经放疗后再行评估入组。在评估时，需关注所采用的评估方式，如按照症状评估的无症状脑转移、放疗后病变稳定脑转移和有症状脑转移，或按照转移数量评估的脑转移。现场核查时需注意对颅脑磁共振成像（magnetic resonance imaging, MRI）的检查结果、既往治疗记录进行溯源，关注受试者在入组时是否存在脑转移或经过放疗的情况，脑转移的评估是否采用方案规定的评估方式等。

#### 2.1.3 既往治疗

肺癌的常规治疗方法较多，包括手术治疗、放疗、化疗、靶向药物治疗等。受试者的既往治疗情况是影响入组临床试验的重要因素。药审中心在指导原则^[3]^中提出，记录所有试验中全部患者的肿瘤治疗史并形成正式文件，为患者符合入组条件提供书面依据。现场核查时建议关注受试者既往就诊记录、诊断证明、影像学报告、病理报告、基因检测报告等以及各类报告之间的时间逻辑关系。受试者既往接受抗肿瘤治疗的证据是否支持入选标准中对治疗线数或复发/难治的要求，是现场核查中应考虑关注的重点。对于要求入选初治患者的临床试验，特别是开放标签试验，或诊断时间与签署知情同意书时间间隔较长的入组受试者，仅以受试者主诉作为既往无抗肿瘤治疗的依据是不充分的。

#### 2.1.4 实验室检查异常

肺癌晚期受试者本身可能经过多次抗肿瘤治疗，身体条件欠佳，或伴有多种有临床意义的实验室检查异常。在筛选时可能存在因骨髓储备或肝肾器官功能不足、病毒学检测结果异常、既往治疗毒性未恢复等影响入组的情况。现场核查时建议重点考虑研究者对实验室检查异常值判定的及时性与合理性，确认受试者入组时的身体状态符合方案的入选与排除标准。如关注研究者是否对筛选期实验室检查异常值进行了判断，在受试者接受试验药物后是否出现了与该异常值有关的安全性问题，是否存在漏做检查或检查报告尚未出具时已经将受试者入组试验的情况等。

#### 2.1.5 筛选失败受试者

受试者在筛选期可能出现筛选失败的情况。与随机对照试验相比，单臂试验设计产生偏倚的风险更高。对于单臂试验，药审中心要求在注册资料中提交筛选失败的受试者清单并说明筛选失败原因。现场核查时应对筛败原因逐一核实，核查保留的源文件是否支持受试者筛选失败。例如，某受试者筛败原因为脑转移放疗后脑水肿，则需查看支持脑水肿诊断的影像学报告等相关证据。对于筛选失败后进行重复筛选的受试者，需关注此前筛选失败的原因以及再次筛选时（如试验方案允许）是否已满足入选与排除标准。

### 2.2 疗效评估方面

药品注册核查的核心思维是基于风险的证据链思维。随着药物研发阶段的不断推进，越到后期的数据越关键^[4]^。支持不同疗效终点的数据质量直接影响试验结果甚至试验成败。因此在核查试验药物疗效的关键数据时，应严格遵循可归因性、易读性、同时性、原始性、准确性、完整性、一致性和持久性的数据完整性标准（attributable legible contemporaneous original accurate complete consistent enduring available, ALCOA+）原则。

#### 2.2.1 OS终点

生存期是迄今为止评价抗肿瘤药物最可靠的临床试验终点。当研究能充分评价生存期时，它通常是首选终点^[5]^。OS定义为从随机化开始到因各种原因导致患者死亡的时间。此终点精确可测，并有死亡日期提供依据，在终点评估时不会出现偏倚。根据临床实际，多数肿瘤晚期患者在临终时都选择返回家中，研究者需在受试者家属的配合下获取受试者死亡时间证明，存在一定难度。研究者作为确保临床试验数据符合“ALCOA+”的责任主体，应采取措施保证生存期数据的收集符合要求。申办者应当识别影响到临床试验关键数据的风险，对受试者死亡日期数据的收集和记录制定相关标准操作规程，确保临床试验数据的可靠性。在现场核查中，建议关注受试者死亡确切日期的溯源，查看受试者死亡证明、殡葬证明、户口注销证明、研究者记录的访视记录等资料，核对死亡日期数据的获取是否满足“ALCOA+”的原则。

#### 2.2.2 基于肿瘤测量的终点

基于肿瘤测量的终点指标主要指ORR、PFS、疾病进展时间（time-to-progression, TTP）、和治疗失败时间（time to treatment failure, TTF）等。胸部增强计算机断层扫描（computed tomography, CT）、上腹部增强CT或B超、头部增强MRI（或增强CT）以及全身骨扫描，是肺癌分期和诊断的主要方法，也是肿瘤评估的主要测量手段。影像检查及评估过程的差异可能会导致测量误差增加、试验终点评估变异增大，最终影响试验结果，因此通常由对研究治疗分配处于盲态的独立终点审查委员会（Independent Review Committee, IRC）验证该终点指标的评价。

现场核查中发现，越来越多的临床试验将IRC评估结果作为主要终点。作为抗肿瘤药物疗效评估的重要数据来源，IRC评估结果的数据可靠性尤为重要。现场核查中建议关注是否确保了多个评估者进行判断的独立性，是否存在数个评估者以会议形式共同对影像数据进行评估的情况；IRC评估人员是否具备章程规定的相应资质；评估流程和仲裁流程与章程规定是否一致等。此外，建议重点对评估结果的真实性和可溯源性开展核查，关注评估人员独立评估结果的可溯源性，评估结果的仲裁是否符合章程规定等内容。

### 2.3 安全性数据记录和报告方面

安全性数据记录和报告的规范性和完整性是评价药物安全性的基础，也是整个研发环节提高和改善药物安全性的重要基石。现场核查时需通过比对试验源文件和源数据，查看不良事件（adverse event, AE）是否被如实记录，从AE的例数、严重程度、与试验药物相关性的判断、是否导致受试者停药或脱落、持续时间、转归情况等多个方面核查药物安全性数据的可靠性。

#### 2.3.1 AE记录和报告的完整性

靶向药物和免疫治疗药物已知的不良反应较多。不良反应不仅会影响受试者依从性，还可能导致受试者药物减量或停药，从而影响疗效评价或使受试者失去治疗机会。研究者应充分关注受试者接受治疗后出现的身体不适，及时作为AE进行记录并报告，确保AE的记录和报告的完整性。在现场核查中建议重点关注临床试验中是否存在AE漏记的情况，特别是对于头痛、心慌、恶心、乏力、皮肤出血等受试者主诉的AE，应对照病历、日记卡、问卷等原始记录核对源数据与病例报告表的一致性。

#### 2.3.2 免疫相关不良事件（immune-related AE, irAE）的鉴别与判断

目前免疫治疗药物已经可以应用于晚期肺癌的一线治疗，如免疫检查点抑制剂。此类药物的常见不良反应表现为各种免疫性炎症等，尤其是具有致死性的心肌炎、免疫性肺炎等需要重视^[6]^。临床试验方案通常要求研究者对发生的AE是否与免疫相关进行判断。因此是否为irAE的鉴别诊断直接影响AE与药物的相关性判断。例如，对可明确诊断为免疫性肺炎（出现胸闷气喘症状、痰培养血培养结果阴性、经验性抗感染治疗症状未好转、治疗期间肺部肿瘤缩小排除疾病进展所致、使用激素冲击治疗后好转）的受试者，应判断为irAE且与试验药物相关。药审中心发布的相关指导原则^[7]^中明确irAE属于不良反应（adverse drug reaction, ADR）范畴。对于存在时间相关性且不存在其他重要混杂因素的罕见严重事件，也常被判定为ADR，如Stevens-Johnson综合征（Stevens-Johnson Syndrome, SJS）和中毒性表皮坏死解松解症（toxic epidermal necrosis, TEN）。现场核查中建议对irAE的记录、报告和判定的准确性与合理性予以充分关注，并对irAE出现后受试者是否得到充分的救治或在给药前是否给予与试验方案一致的预防治疗等情况进行核查。

#### 2.3.3 AE与试验药物的相关性

验证性临床试验的目的之一，是在药物上市前尽量充分暴露药物的ADR，以指导临床合理用药。试验方案中会规定AE与试验药物相关性判定的基本原则，并经判定后获取ADR数据。临床试验中存在AE与试验药物相关性判断与试验方案规定的判定原则不符的情况。现场核查时建议关注研究者对AE与试验药物相关性的评估是否遵循方案规定的判定原则，特别是试验药物已知的ADR和irAE。研究者应本着对未知的尊重，以对试验、受试者和目标患者负责任的态度，充分考虑用药与AE出现的时间关系、是否符合该药已知不良反应类型、去激发或再激发结果、是否有其他原因可以解释等因素，科学严谨地判定相关性。

### 2.4 试验用药品管理方面

肺癌靶向治疗药物中，小分子TKI类药物为口服制剂，稳定性较好，说明书一般要求30 ℃以下保存，在临床试验中给予试验药物时受试者的依从性较好，药品运输、储存条件较为宽松。免疫治疗药物多为注射给药，需严格按照说明书或试验方案要求进行储存、运输、配置和输注。如信迪利单抗要求将药瓶于2 ℃-8 ℃的冷藏环境下保存在原包装中，避光、避免冷冻、避免震荡，使用前将药瓶恢复至室温（25 ℃或以下），稀释前可在室温下最长放置24 h，一经稀释必须立即使用，不得冷冻。给药前应目测注射用药是否存在悬浮颗粒和变色的情况，如观察到可见颗粒，应丢弃药瓶。输注时所采用的输液管必须配有一个无菌、无热源、低蛋白结合的输液管过滤器（孔径0.2 μm）。输液时间在30 min-60 min内。研究者和申办者应做好临床试验启动前的培训，加强试验各环节的过程管理，及时发现问题并采取纠正预防措施，确保临床试验中的药品管理过程符合试验方案要求。在现场核查中需关注试验药物的运输、储存是否符合方案或说明书规定，并根据配置或输注的原始记录核对其与方案或说明书相关要求的一致性。

## 3 讨论

抗肿瘤药物试验设计复杂，可能产生的数据质量风险为临床核查带来挑战。由于肿瘤的复杂性，不同机制、不同靶点的药物联合治疗仍然是提高疗效和克服耐药的重要手段。目前两个或多个新药联合治疗，或多个新药与标准治疗联合的试验设计层出不穷。由于联合治疗可能增加受试者的风险，试验设计除关注增效证据外，还需特别关注药物安全性风险的预防、识别、监测和干预。此外，单臂试验或开放标签设计对药物疗效和安全性的评估可能产生偏倚；适应性设计对方案执行的依从性以及数据管理和统计分析提出更高要求；真实世界研究的数据治理过程应满足完整、准确、透明的原则；去中心化临床试验可能采取的远程访视、远程实验室评估、远程成像评估、远程监测、居家随访等新模式所带来的新的数据质量风险等，均给临床核查带来了诸多挑战。从核查的角度，应继续讨论不同试验设计中可能导致数据质量风险的环节，从而基于风险进一步分析研判核查的关注点。

临床研究者作为临床试验的执行人，对临床试验的质量起着非常关键的作用，也是注册临床核查的重点对象。基于对临床试验数据的真实性、一致性和可溯源性进行确认的核查目的，目前我国的药品注册临床核查以对研究者/临床试验机构的核查为主。在美国食品药品监督管理局（Food and Drug Administration, FDA）开展的生物学研究监测项目（Bioresearch Monitoring Program, BIMO）中，临床研究者接受官方检查的数量也排在首位，远超其他检查对象。此外，在临床试验全过程中，数据管理、统计分析、监查稽查等也是影响试验质量和试验结果的重要环节。探讨对发生在临床试验机构以外的试验数据处理过程的核查是进一步提升药品注册临床核查质量的方向之一。

检查员不仅应具备发现问题的能力，还需具备判断问题性质、责任方的能力。在欧盟的药物临床试验检查中，检查报告需对每一条发现问题的严重程度和责任方进行明确。对核查发现问题的严重程度分级有助于指导和帮助检查员对发现问题的严重程度进行综合判断。对问题的责任方进行明确，有助于各责任方在各自的职责范围内及时整改。

## 4 展望

随着靶向药物、免疫治疗的飞速发展，肺癌治疗药物临床试验日益升温。在临床诊疗指南中提到，参加临床试验已经作为患者疾病进展后的一种临床治疗选择。临床试验实施的质量不仅影响试验结果和试验数据的可靠性，更关系到受试者的生命和权益。研究者和申办者应牢固树立质量意识和质量源于设计的理念，充分理解数据可靠性的要求，对数据产生过程进行质量监控，及时查找和发现风险，确定其影响范围，采取纠正措施，必要时向药品监管机构报告，共同讨论解决存在的问题^[8]^。建议在临床核查中围绕以患者为核心的理念，积极探讨基于不同适应证或不同试验设计的核查关注点，探索基于风险的临床核查模式，提高临床核查的质量和效率。希望通过研究者、申办者和监管部门的共同努力，确保临床试验实施的整体质量，确保试验数据的真实可靠，确保受试者安全权益得到合理的保护，促进我国创新药的研发。
